# Investigating Numerically the Effect of Wind on Fire Spread Between Two Informal Settlements Dwellings

**DOI:** 10.1007/s10694-023-01374-y

**Published:** 2023-03-06

**Authors:** M. Beshir, M. Mohamed, S. A. Kouritem, C. K. Lemmertz, F. R. Centeno, D. Rush

**Affiliations:** 1 School of Engineering, University of Edinburgh, Edinburgh, UK; 2 OFR Consultants, Sevendale House, Lever St., Manchester, M1 1JA UK; 3 School of Ecosystem and Forest Sciences, University of Melbourne, 4 Water St., Creswick, VIC 3363 Australia; 4 Mechanical Engineering Department, Faculty of Engineering, Alexandria University, Alexandria, 21544 Egypt; 5 School of Engineering, Federal University of Rio Grande do Sul, Porto Alegre, RS 90050-170 Brazil

**Keywords:** Informal settlements (IS), Fire dynamics, Compartment fires, Computational fluid dynamics, Fire dynamics simulator (FDS), Wind effects

## Abstract

Previous full-scale fire studies revealed that the role of wind on fire spread between informal settlement dwellings was critical. However, the influence of wind conditions on informal settlement dwellings fire spread is currently understudied in the literature. This study aimed to investigate the effect of external wind conditions on fire spread between two informal settlement dwellings with a distance of 1 meter between them. A parametric numerical analysis was performed using the computational fluid dynamics code Fire Dynamics Simulator. The numerical models were benchmarked through laboratory experiments. The investigation included an analysis of the fire spread mechanism, flashover conditions, and heat transfer processes at the boundaries of the dwellings. Simulations were conducted with burning wood cribs as fuel and three wind speeds (6 m/s, 10 m/s, and 14 m/s) with four wind directions (East, West, South, and North). Results showed that wind speed and direction had a significant impact on the fire dynamics of the origin dwelling and its spread to neighboring dwellings. The wind direction also influenced the time to flashover in both dwellings, with a delay observed when the wind flowed through the alley between the two dwellings. The total heat transfer coefficient was found to be directly proportional to the wind speed for all directions. The internal radiative heat transfer coefficient of one wall was found to represent the total heat transfer coefficient in different scenarios. This study highlights the complexity of determining the role of wind in urban fire spread and underscores the need for further research in this area.

## Introduction

As one of the most pressing issues of the twenty-first century, urbanisation requires careful planning and attention to such factors as housing, infrastructure, and public security [1]. It is estimated that an annual migration of 70 million individuals into metropolitan regions takes place [2], with much of this increase happening in the global south during the previous several decades. As urban populations expanded, an increasing number of people were compelled to reside in inadequate informal settlements (the term informal settlement covers all the settlements that do not have legal state permission to exist [3]). In South Africa, for instance, informal settlements (IS) account for roughly 13% of all households, or about 2.19 million out of 16.9 million [4], whereas in Brazil, it is estimated that over 11 million people, or about 6% of the entire population, live in IS [5]. In informal settlements, fires often move from one dwelling to another by heat flux or flame impingement from a dwelling that has attained flashover [6]. South African houses in informal settlements typically consist of a timber frame covered in either steel sheets or wooden boards [7]. Fire loads within informal settlement dwellings (ISD) near Stellenbosch, South Africa were determined to be about 410 MJ/m^2^ (with a standard deviation of 140 MJ/m^2^), according to a preliminary study [8]. However, fire loads in certain residences might reach up to 2000 MJ/m^2^ (e.g., if storing fuel such as wood, paraffin or gas bottles). Imizamo Yethu, a township in Cape Town, South Africa, was the site of one of the most recent devastating fires in an informal community. In addition to causing 4 fatalities and injuring 2 firemen, this fire also destroyed 2194 buildings [9]. A survey of the firefighters who responded to the fire found that the velocity and direction of the wind greatly facilitated the fire’s capacity to disseminate throughout the settlement. Wind's influence on the fire's progress in Imizamo Yethu was also studied by Kahanji et al. [9], who calculated that 75% to 85% fewer homes would have been destroyed if the wind hadn't been shifted during the fire due to wind. Currently, there are many efforts aiming to understand some of the fire risks within IS through different scale experimentation [6, 8, 10–12] and numerical modelling [13–15] with the purpose of providing the necessary basis and future research directions to make cities more inclusive, safe, resilient and sustainable. Koker et al. [16] conducted a 20-dwelling large-scale fire spread experiment for ISD in 2020 to determine the various modes of fire spread between dwellings. The results indicated that the proximity between dwellings (i.e., 1 m to 2 m) and the wind speed and direction played the most significant roles in directing and driving the spread. Even with the moderate wind speed on the day of the experiment (4 m/s to 7 m/s), the fire spread over the entire settlement in around five minutes. In addition, most of the structures reached flashover within a minute. This experiment revealed that the behaviour of fire spread in IS can be quite similar to that of wildfires, with a continuous fire front spreading around the area at slower spread rates. Assessing the influence of wind on compartment fires can be challenging, however numerical simulations have proven to be a useful method, especially when full-scale experiments are infeasible due to their size or the required time and resources. The bulk of published fire simulation research have used the computational fluid dynamics (CFD) code fire dynamics simulator (FDS) [17]. Centeno et al. [18] numerically studied thermally-thin and thermally-thick bounded reduced scale compartment fires in front of a wind tunnel. By changing wind speed and direction, the study investigated how wind affects the compartment's heat release rate (HRR) needed to reach flashover conditions (${\dot{q}}_{fo}$). Since it assumed a prescribed HRR, this study did not account for the wind's effect on burning rate enhancement. It was found that, the wind speed and direction strongly affect the ${\dot{q}}_{fo}$ for both walls’ boundary conditions. The induced pressure profile around the compartment's opening and heat transfer through the walls are the driving mechanisms for thermally-thick walled compartments and thermally-thin bounded compartments, respectively. Lemmertz et al. [19] conducted a numerical study on a single full-scale ISO-9705 compartment fire to understand the influence of the wind conditions as speed and direction (side and back of the dwelling only) and wall thermal characteristics on the time to reach the onset of flashover, fire dynamics and fire spread in informal settlements. It was observed that regardless the wind direction and boundary characteristics, the increase in wind speed considerably reduced the time needed to reach flashover. They concluded that this happens due to the wind enhancing the burning rate of the wood cribs, and consequently accelerating the hot gas temperature rise. The increase in wind speed also accelerated the occurrence of flashover and hence flame ejection through the door happened earlier. The radiative heat fluxes measured outside the door also increased with the increase in wind speed. They concluded that both wind speed and wall thermal characteristics significantly affect the severity of informal settlement fires and can increase the risk of fire spread, recommending the investigation of the wind effect on the fire spread between dwellings. Considering this backdrop, this study used the CFD model FDS to investigate quantitatively the influence of wind on internal fire dynamics and the mechanism of fire spread between two close ISDs. Wang et al. [6] conducted fire spread experiment between two ISD that were separated by 1.0 m. The experiment used to benchmark the FDS model was conducted in a laboratory (environment with still air) with sophisticated apparatus in both compartments to quantify HRR, temperatures, and heat fluxes. Thus, the current study will benchmark a FDS model using Wang et al.'s fire experiment [6], and then use the model to numerically examine the effect of different wind conditions on the fire dynamics and fire spread between two ISD.

## Numerical Model (FDS)

Fire Dynamics Simulator (FDS version 6.7.6) [17] was used as a numerical tool in this study. FDS has been developed by the national institute of standards and technology (NIST) for solving a set of Navier–Stokes equations via a second-order finite difference numerical scheme appropriate for low-Mach number flows.

### Turbulence Modelling

FDS adapts the large eddy simulation (LES) modelling technique, where the turbulence model represents the closure of the sub grid Scale (SGS) flux terms with the eddy viscosity term being of the most crucial variable. Thus, FDS provides several models to solve the turbulent viscosity parameter, with the modified Deardorff model [20] set as the default sub-grid scale turbulent viscosity solver which will be used for all the simulations in this work.

### Combustion Modelling

The default combustion model in FDS is based on the mixing-limited, infinitely fast reaction of lumped species [17]. FDS uses a "turbulent batch reactor" comprehensive model, which is developed to account for complex reactions where the mixing and reaction time scales overlap [21]. Each computational cell behaves as a turbulent batch reactor. However, for non-premixed diffusion flames associated with fast chemistry where the kinetics dominates over the rate of mixing, the reactor model reduces to a simple "mixed is burnt" approximation called the eddy dissipation concept (EDC) [22, 23].

### Radiation Modelling

Due to the uncertainties that associate the calculations of flame temperature in cases where the flame cannot be accurately determined on a comparatively coarse computational mesh grid, the source term in the radiation transport equation (RTE), because of its T^4^ dependence, cannot be resolved. Therefore, in FDS, instead of solving the RTE, the value of the radiative fraction can be explicitly specified which is the fraction of the total combustion energy that is released in the form of thermal radiation, and is a function of both the flame size, flame temperature and the composition of chemical species.

### Wind Modelling

FDS provides three different approaches to model wind as follows: (a) Monin–Obukhov Similarity; (b) Letting the Wind Develop Naturally; (c) The Wall of Wind. In this study, the Monin–Obukhov Similarity method is selected and applied for all the cases as it provides an applicable and efficient way to set the wind conditions separately, namely the speed and direction, in addition to the ability of varying them temporally as a function of time. The similarity describes the relationship of the vertical behaviour of non-dimensional mean flow (wind speed profile) and mean temperature (potential temperature) in the atmospheric surface layer. In FDS, the Monin–Obukhov similarity method simulates the wind conditions by specifying the values of wind speed and direction besides the values of Obukhov length scale (L) and aerodynamic roughness length (Z_**0**_).

Accurate knowledge of aerodynamic characteristics of cities is vital to describe, model, and forecast the behaviour of urban wind and turbulence at all scales [24]. The Obukhov length (L) [25] is defined from the frictional velocity and the buoyancy flux from a dimensional analysis. However, due to the difficulty to calculate L analytically, the Obukhov length (L) is often estimated from routine meteorological measurements [26]. The dimensionless height parameter (Z**/**L) is traditionally chosen as the stability parameter, where z is the height above the ground and L is the Obukhov length scale that characterizes the thermal stability of the atmosphere. When L is negative, the atmospheric surface layer is statically unstable stratified, and when L is positive, the atmospheric surface layer is statically stable stratified.

On the other hand, the aerodynamic roughness length (Z_0_) is the theoretical height above the displacement plane at which the mean wind speed becomes zero when extrapolating the logarithmic wind speed profile downward through the surface layer. It is an important parameter for surface fluxes estimates [27]. Consequently, precise mapping of Z_0_ in urban areas is crucial for different modelling applications and for planning purposes [28]. Mainly, the aerodynamic roughness length depends on the geometric features and distributions of the roughness elements [29]. For a glacier zone, Z_0_ is an important control on the rate of turbulent heat transfer between a glacier surface and the air above it [30], while for vegetated surfaces, the mean canopy height, the canopy structure and the plant density are key variables affecting Z_0_ [31]. A description of the aerodynamic roughness length for different landscapes is provided by Davenport-Wieringa roughness length classification. Based on sensitivity analysis, Lemmertz et al. [19] have found that the Obukhov length (L) and the aerodynamic roughness length (Z_0_) have a very little effect on the time required for flashover and the trends in the results were kept the same. However, the results are less sensitive to the Obukhov length than to the aerodynamic roughness length. In this study, the values of L and Z_0_ are taken as suggested by FDS for the specific computational domain being studied to be − 100 m and 0.03 m, respectively. The wind speeds were implemented at a reference height of 10 m above the ground, which is the standard height where the wind parameters are measured in meteorological stations.

## Methodology

The main structure and basis of the present study will be discussed in more detail in the following sections.

### Experimental Set-Up and Results

**Figure 1:**
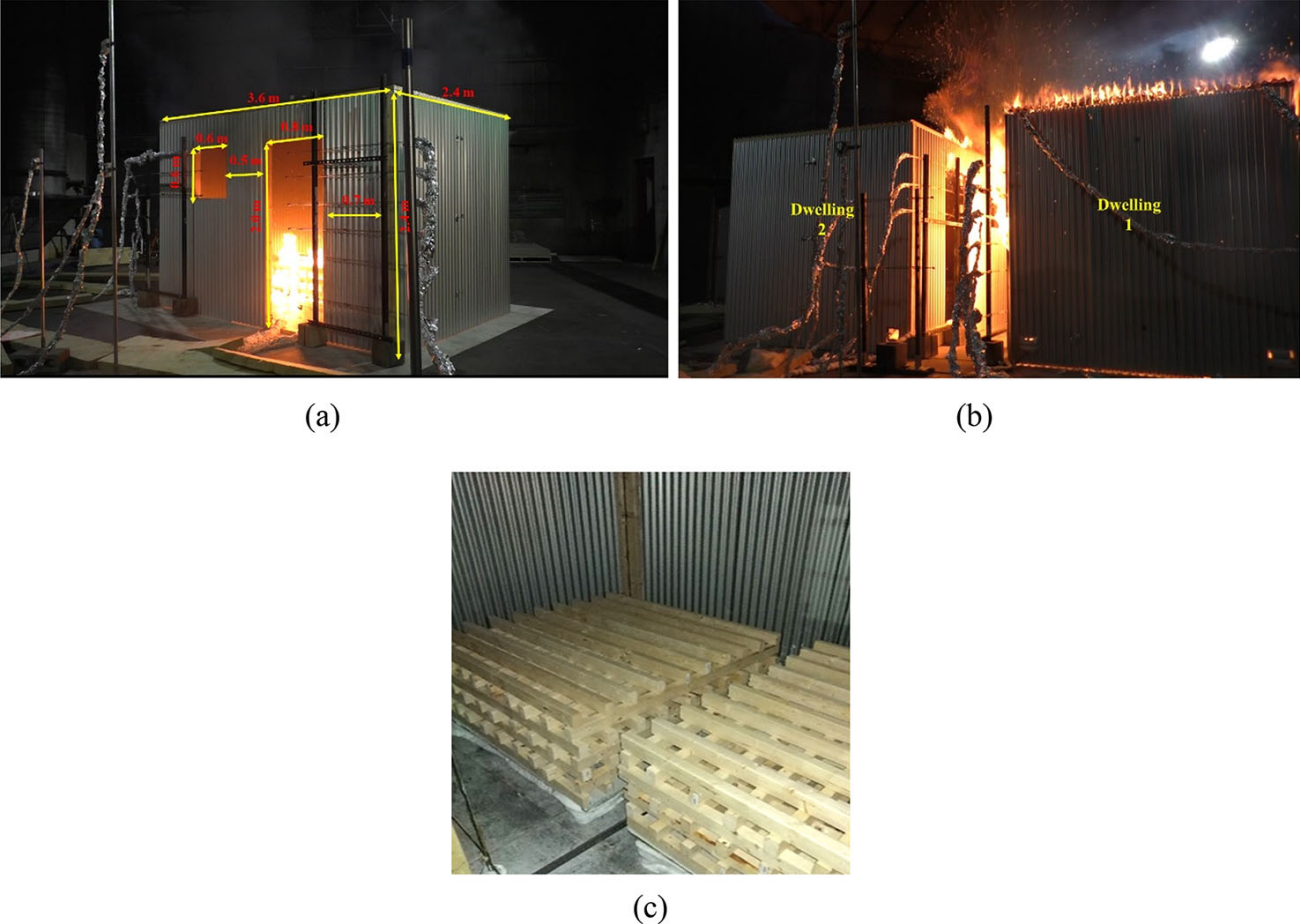
(**a**) single dwelling dimensions, (**b**) Fire spread between the two dwelling experiment [6] and (**c**) wood crib’s configuration used in [6]

In order to investigate the fire development and fire spread mechanisms between ISD, Wang et al. [6] have conducted a fire spread experiment between two ISD separated by 1 m distance, and built with ISO 9705 dimensions of 3.6 m × 2.4 m × 2.4 m as an approximation of an IS dwelling as shown in Figure 1a. Each ISD is designed with a door and window openings on the front long wall and identically opposite to the other ISD as mirrored with internal dimensions of 2.0 m (height) × 0.8 m (width) for the door, and 0.6 m × 0.6 m for the window as presented in Figure 1b. The two ISD were constructed by galvanized steel sheets with a thickness of 0.51 mm, flute height of 22 mm and pitch of 70 mm as the four sidewalls and roof, which were attached to the timber frame with a section of 101.4 mm × 50.8 mm in addition to a cement board with a thickness of 8 mm laid on the floor for insulation. Moreover, two wood cribs were placed in each dwelling at a certain location where each crib consisted of 7 layers of 10 sticks with a dimension of 0.038 × 0.064 × 1.219 m^3^, as presented in Figure 1c, and a wood density of 540 kg/m^3^. Based on a heat of combustion of timber of 17.5 MJ/kg, the fuel load is approximately 437.5 MJ/m^2^, which is roughly close to the average fuel load (410 MJ/m^2^ ± 140 MJ/m^2^) of IS dwellings in South Africa. Dwelling 1 is the fire origin dwelling where the ignition was with ignition source of Gasoline-87 soaked mop head strips in eight plastic bags, where a plastic bag was placed at each crib’s corners. While 4.8 mm thick corrugated cardboard lined the four sidewalls in Dwelling 2 with the ignition of cardboard to be the indication of fire spread between the two dwellings. The results were benchmarked and compared against an independent single dwelling experiment with identical boundary conditions. It was found that the presence of the adjacent dwelling significantly delays flashover and changes the dynamics of the initial dwelling fire, which may be due to experimental uncertainties, and this will be investigated numerically in later sections. Additionally, the ejected flames from the openings were found to play a key role in fire spread, where the fire spread from dwelling to dwelling that is normal to the plane of the openings is significantly concerned with wall collapse or large flame ejections from the burning cardboard linings instead of radiation from high temperature steel sheet wall.

### Numerical Set-Up

#### Numerical Mesh

The computational mesh was generated based on the single-dwelling leaky case in [13]. To create the second dwelling, the fire origin dwelling was mirrored at an offset distance of 1 m from the front openings, and then a cardboard lining was added to the inside surface of the steel-sheets cladding of the second dwelling. The second dwelling was modelling with similar methodology to that used to model the cardboard lined dwelling in [13]. Following the modelling approach in [13], a cell size of 0.06 m was chosen for the whole domain and the fire spread over wood cribs was modelled stick by stick, where this methodology was adopted previously also in [32].

#### Cell Size

The cell size was chosen based on the analysis performed by Beshir et al. [13] according to:

1. Kallada Janardhan and Hostikka [33] suggested a method based on a wood crib adjustable area approach. This method has already demonstrated its effectiveness in reducing computational effort and increasing simulation efficiency by implementing a correction for the mesh dependency of the fuel surface area in cases where the CFD grid cells are larger than the sticks that use the ignition temperature for pyrolysis, allowing for the use of a coarser mesh.
2. Cell-size check using the method proposed by Quintiere [34], namely the D* method, which is a measure of how well the flow field is resolved based on the non-dimensional expression D*/ δx, where D* is the characteristic fire diameter calculated as:
  1 $${\text{D}}^{*} = \left( {\frac{{{\dot{\text{Q}}}}}{{{\uprho }_{\infty } {\text{C}}_{{\text{p}}} {\text{T}}_{\infty } \sqrt {\text{g}} }}} \right)^{2/5}$$

where $\dot{\text{Q}}$ is the total heat release rate of fire (HRR), ${\uprho }_{\infty }$ is the ambient air density, ${\text{T}}_{\infty }$ is the ambient air temperature, ${\text{C}}_{\text{P}}$ is the specific heat of air, g is the gravitational acceleration, and δx is the cell size. The quantity D*/δx can be thought of as the number of computational cells spanning the characteristic (not necessarily the physical) diameter of the fire. Obviously, the more cells spanning the fire, the better the resolution of the calculation. Based on the D* the cell size to be used in the simulations, should be less than 0.1 D*. Assuming a HRR of around 3000 kW, the recommended cell size will be around 26 cm where the cell size used in these simulations is 6 cm (i.e., around four times smaller). The D* also showed good performance with wood cribs modelling as reported by other researchers [7, 13, 35, 36]

#### Simple Pyrolysis Model

The HRR per unit area (HRRPUA) of the wood (soft pine wood) and cardboard were taken from the previous cone calorimeter study [37] under the heat flux of 75 kW/m^2^ for both materials. The ignition temperature of the wood and the cardboard was set to be 250 °C and 350 °C, respectively. The heat of combustion was taken as 20 MJ/kg [38] for the dominating fuel (Pine wood). The chemical composition of the wood was set as input for the FDS as C_**3.4**_H_**6.2**_O_**2.5**_ [39] with a soot yield of 0.015 [40]. The accelerant/igniters (gasoline)’s HRRPUA curve used in the tests, was based on the early stage HRR curve of the tests in [41], where a sensitivity analysis for these inputs has been discussed by Cicione et al. [42].

#### Thermal and Physical Properties of Materials

The wood’s bulk density was chosen as 535 kg/m^3^ [38], which was adapted to 455 kg/m^3^ based on the cell size according to the method proposed by Kallada Janardhan and Hostikka [33], while the specific heat was assumed to be 1.3 J/(g K), and the conductivity to be 0.2 W/(m K). The steel was assumed to have a specific heat of 0.6 J/(g K), conductivity of 45 W/(m K), density of 7850 kg/m^3^, and emissivity of 0.2 (new shiny galvanized steel sheets) [43].

#### 1-D Conduction Heat Transfer

The steel sheets were of thickness of 0.5 mm which is also the thickness of the 1-D heat transfer model set in the current simulations. Since the steel walls thickness (0.5 mm) was much less than the cell size, therefore, the walls back side condition was set as EXPOSED FDS command for the 1-D heat transfer interaction between the two sides of the wall.

#### The Instrumentation

**Figure 2:**
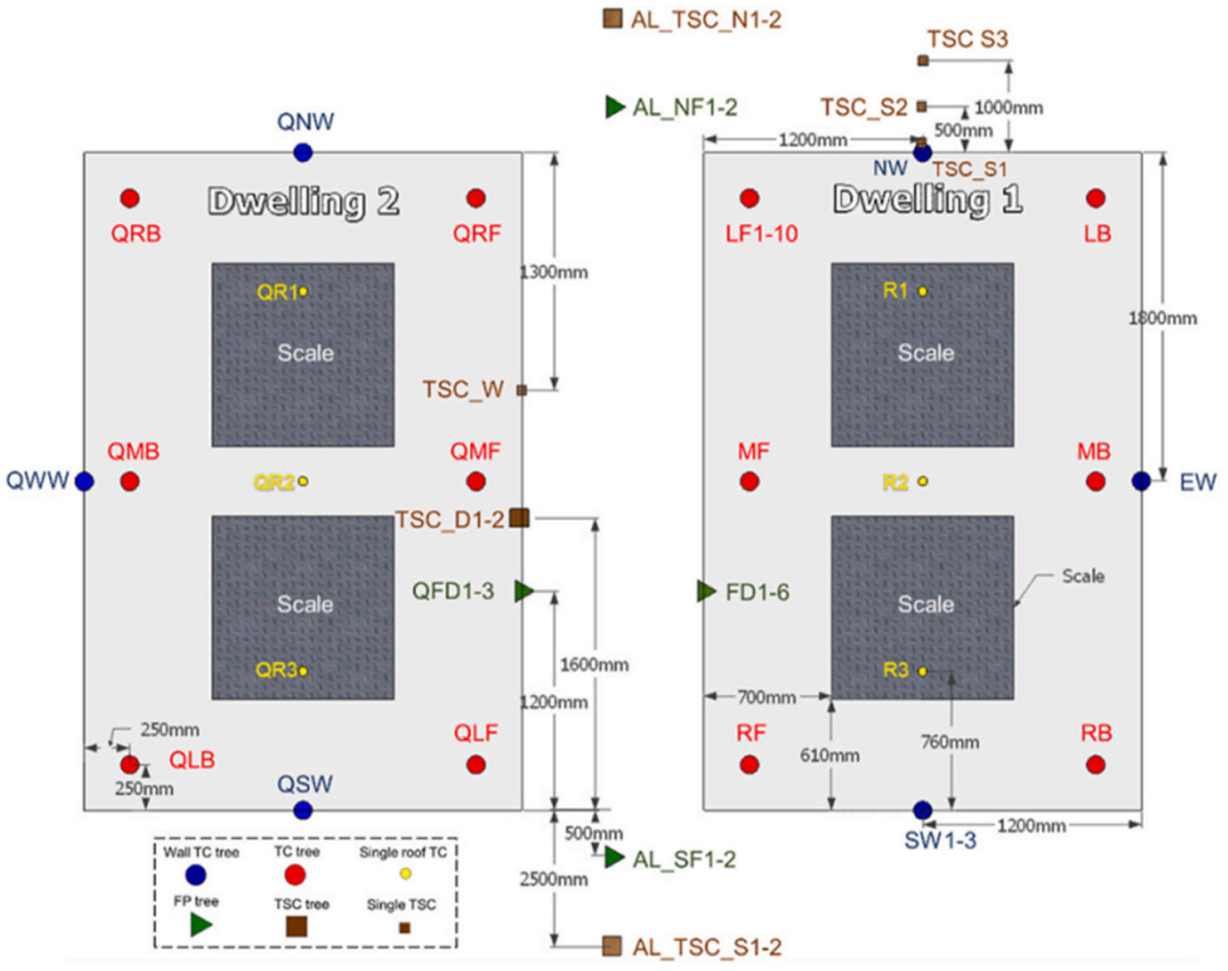
Instruments distribution (*TC* thermocouple, *TSC* thin skin calorimeter, *FP* flow probe) from [6]

Same instrumentations as that used in [6] were modelled in this study, as presented in Figure 2. The thermocouple trees within the compartment were simulated using thermocouples of the same bead diameter (1.0 mm). The flow velocities were measured using a device with the quantity v-velocity, the TSCs were simulated using the device with the quantity radiative heat flux gas and the wall temperatures were measured using the wall and back wall temperature devices. Additionally, the gas concentrations were measured with gas analyzers for the O_**2**_ and CO_**2**_ at the same locations for the experiments.

#### Constant Wind Conditions

**Figure 3:**
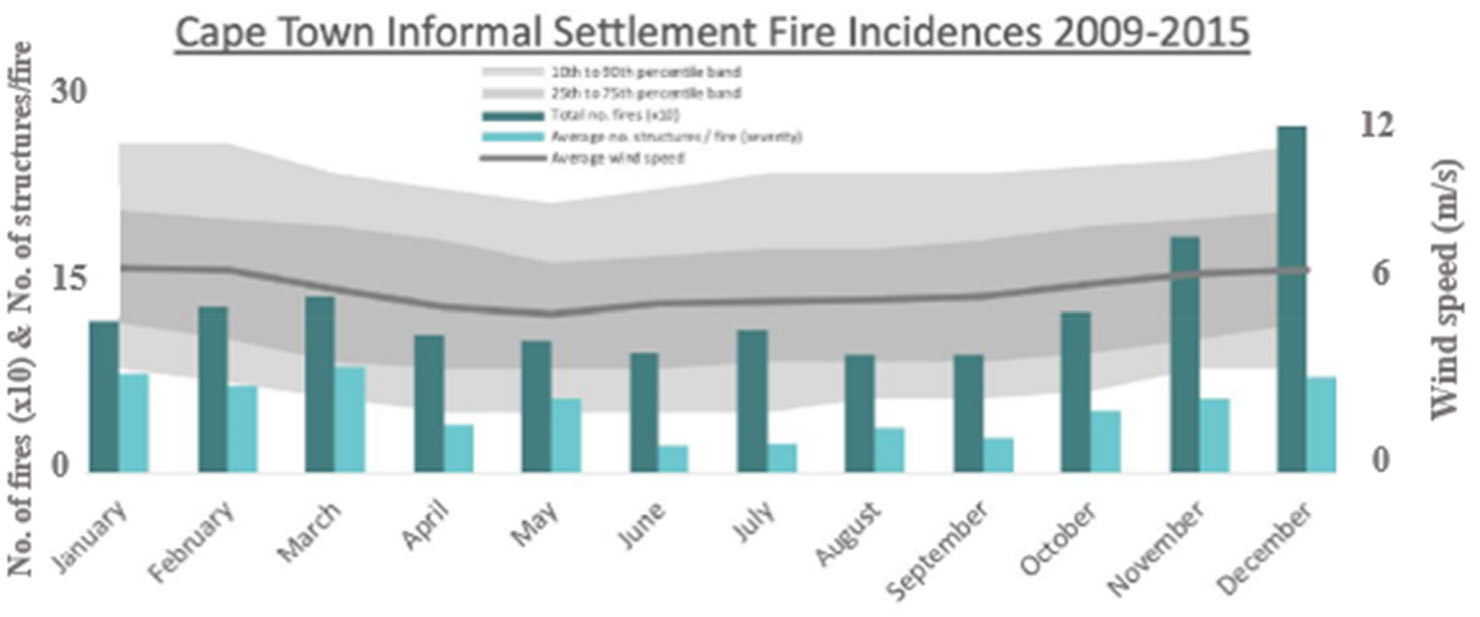
Maximum wind speed in every month from 2009 to 2015 in Cape Town (m/s) [44]

**Figure 4:**
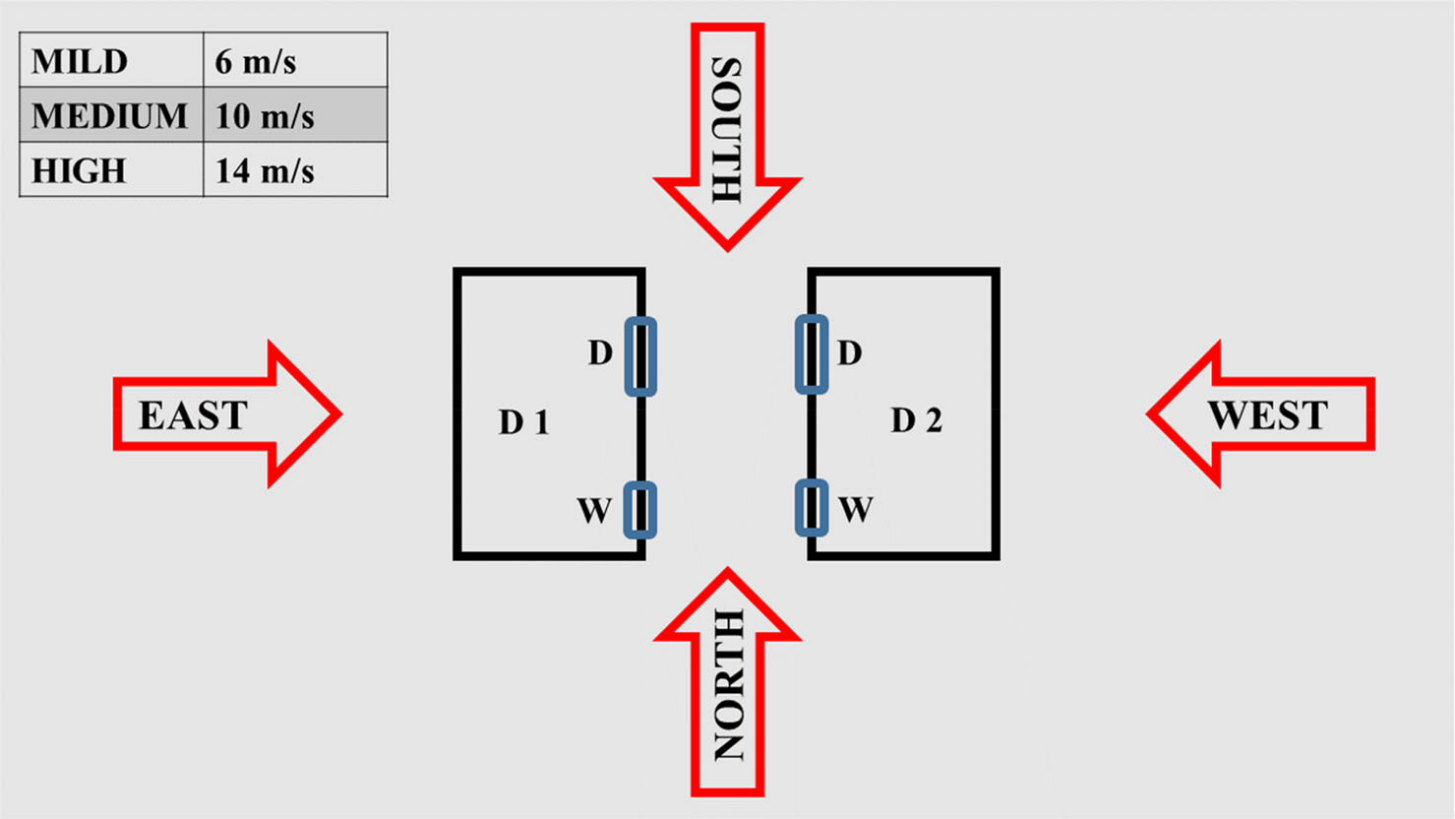
Wind speeds and directions, where D stands for door and W stands for Window

A wind flow field will be incorporated in the numerical baseline fire model (no wind conditions [6]) in order to examine the wind effect on the fire growth and spread between the two dwellings under various wind conditions. According to prior research [9, 18] the environment parameters influencing fire propagation include both wind speed and wind direction. Wind speed: input parameter for the FDS model is derived from the wind database in Cape Town from South Africa Weather Service (Cape Town International Airport) as presented in Figure 3. Through available calculations from [44], the possible maximum wind speed during the period between 2009 and 2015 years is up to around 12 m/s to 13 m/s, and the data of average wind speed in every month in this period in Cape Town, shows that the average wind speed of these years is about 6 m/s to 5 m/s. Therefore, three wind speed values are selected to be within this specified range: (a) 6 m/s (mild); (b) 10 m/s (moderate); (c) 14 m/s (severe). Wind directions: will account for a total number of four wind directions to be tested. Assuming that the wind is blowing to the four cardinal directions, which are North (N), East (E), South (S), and West (W). For each wind direction, three wind speeds (e.g., mild, moderate, and severe) will be explored. The distribution of the wind directions is represented by the schematic diagram in Figure 4. The modelling scenarios in this study will investigate the combination of the four different wind directions and three wind speeds, resulting in a total of 12 scenarios. It is important to recall that, the implementation of the fire scenarios on FDS followed strictly the specifications of the experiment in [6].

### Theoretical Heat Transfer Analysis

As indicated by previous studies [13, 14, 45], the radiative emission and absorption processes between the hot gas layer (including soot aggregates and smoke) and the thermally-thin walls/boundaries of ISD highly affects the onset of flashover, besides featuring prominently when analyzing the heat transfer mechanisms to both of the surroundings and fuel package. In the current study, a heat transfer analysis will be implemented, seeking to numerically understand the influence of wind on the spatial and transient temperatures of the wall boundaries, the gas-layer and the surrounding of a thermally-thin bounded ISD which is subjected to fire spread from neighbouring dwelling in the presence of wind. All inclusive, the thermal resistances by conduction through the boundary (R_cond_), convection on/from the internal and external surfaces of the boundary (R_conv_), and radiation on/from the internal and external surfaces of the boundary (R_rad_), the overall heat transfer coefficient (U) can be defined as:

2 $${\text{U}} = {\text{R}}_{{{\text{cond}}}} + {\text{R}}_{{{\text{eq}},{\text{int}}}} + {\text{R}}_{{{\text{eq}},{\text{ext}}}} = \left( {\frac{\delta }{{\text{K}}} + \left( {\frac{1}{{{\text{h}}_{{{\text{conv}}}} + {\text{h}}_{{{\text{rad}}}} }}} \right)_{{{\text{int}}}} + \left( {\frac{1}{{{\text{h}}_{{{\text{conv}}}} + {\text{h}}_{{{\text{rad}}}} }}} \right)_{{{\text{ext}}}} } \right)^{{ - 1}}$$

where δ is the wall thickness, K is the wall thermal conductivity, h_conv_ is the convective heat transfer coefficient, and h_rad_ is the radiative heat transfer coefficient.

The radiative heat transfer coefficient (h_rad_) is computed as follows:

3 $${\text{h}}_{{{\text{rad}}}} = \varepsilon \sigma \left( {{\text{T}}_{{\text{B}}}^{2} + {\text{T}}_{{\text{S}}}^{2} } \right)\left( {{\text{T}}_{{\text{B}}} + {\text{T}}_{{\text{S}}} } \right)$$

where ε is the surface emissivity, σ is the Stefan–Boltzmann constant (5.67 × 10^–8^ W/(m^2^ K^4^), T_B_ is the temperature of the boundary/wall and T_S_ is the temperature of the surroundings.

The free convective heat transfer coefficient (h_conv_) is computed using the Nusselt (Nu) number correlation for laminar free convection for both internal and external surfaces as follow:

4 $${\text{Nu}} = \frac{{{\text{h}}_{{{\text{free}},{\text{conv}}}} {\text{ H}}}}{{\text{K}}} = 0.68 + \frac{{0.67{\text{ Ra}}_{{\text{H}}}^{1/4} }}{{\left( {1 + \left( {0.492/{\text{Pr}}} \right)^{{{\raise0.7ex\hbox{$9$} \!\mathord{\left/ {\vphantom {9 {16}}}\right.\kern-0pt} \!\lower0.7ex\hbox{${16}$}}}} } \right)^{4/9} }}$$

where H is the wall height, K is the gas thermal conductivity, Pr is the Prandtl number, and Ra_H_ is the Rayleigh number given by:

5 $${\text{Ra}}_{{{\text{H}}}} = \frac{{{\text{g}}\beta \left( {{\text{T}}_{{\text{B}}} - {\text{T}}_{\infty } } \right){\text{H}}^{3} }}{{{{\nu }}\alpha }}$$

where T_B_ is the wall surface temperature, T_∞_ is the ambient temperature (considered as the hot gas layer temperature when analyzing the internal surfaces and as the ambient temperature when analyzing the external surfaces), β is the thermal expansion coefficient, ν is the kinematic viscosity, α is the thermal diffusivity. The gas thermal properties are taken as of air by averaging over the range between maximum and minimum temperatures [46]. Equation (5) is valid for laminar free convection (Ra_H_ ≤ 10^9^).

The convective heat transfer coefficient (h_conv_) on the external surface must be a combination of a free convection coefficient (Eq. 4) and a forced convection coefficient, assumed here as [47]:

6 $${\text{h}}_{{{\text{forced}},{\text{conv}}}} = 5V_{{wind}}^{{0.85}}$$

where V is the wind speed.

The study will follow the same procedure for computing T_B_ and T_S_ as reported in [14]. It was considered that T_B_ is uniform on the walls (taken as an averaged value for nine points on the wall, three points on the left corner, three on the right corner and three on the middle). The points were equally distributed vertically bottom, middle and top of each section (this is a reasonable consideration, since the walls have low Biot number and the thermal gradient could be ignored), and T_S_ was considered as the hot gas temperature when analysing the internal heat transfer resistance (R_eq,int_) and as the ambient temperature when analysing the external heat transfer resistance (R_eq,ext_). Then, the overall heat transfer coefficient taking into account all boundaries except the ceiling is obtained as follows:

7 $${\text{U}}_{{{\text{tot}}}} = \sum \frac{{{\text{A}}_{{{\text{wall}}}} }}{{{\text{A}}_{{{\text{tot}}}} }}{\text{U}}_{{{\text{wall}}}}$$

where U_wall_ is the wall overall heat transfer coefficient, A_Wall_ is the wall area, and A_T_ is the total area of boundaries.

## Results and Discussion

For the sake of simplicity, in this study the naming of each scenario was based on the wind speed and wind direction (notation; N: North, S: South, E: East and W: West). For example, 6E stands for the wind blowing in the East direction with speed of 6 m/s. The fire origin source, which is dwelling one will be called D1, and dwelling two to where the fire spread will be called D2.

### Numerical model benchmarking

**Figure 5:**
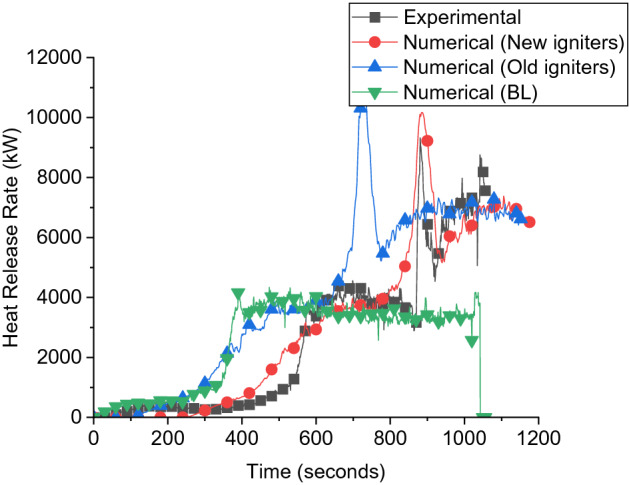
HRR of various numerical models compared to experimental HRR

The first stage in this study is to benchmark the numerical model using the two dwellings experimental work presented earlier by Wang et al. [6]. The benchmarking will be done through comparing the FDS results against the data obtained from the original experiment, namely the HRR, gas layer temperature within the dwelling, oxygen concentration and incident radiative heat flux. The two dwellings in the experimental work [6] were built identically to two single dwellings large scale experiments conducted and presented in [41], where D1 and D2 were built identical to the single dwellings BL and CB, respectively (where BL stands for the base line experiment and CB stands for a scenario similar to the BL but with the internal walls lined with cardboard). In the experimental paper presented by Wang et al. [6], one of the main questions in the experimental results was the delay in the flashover time for dwelling 1 (D1) compared to a similar dwelling namely BL single case presented in [42], this was assumed to come back to human error in ignition or the effect of dwelling 2 (D2) being placed at 1.0 m from the openings and blocking the inlet flow somehow. To investigate this observation numerically, the two dwellings were modelled with the same input used in modelling the baseline (BL) and cardboard (CB) cases single dwellings presented in [13]. Figure 5 presents the HRR-time curve for the Double Dwelling (DD) experiment, BL numerical results case and the numerical model. In the numerical work, D1 was modelled the same way as modelling the BL case. Using these inputs, the flashover of D1 occurs at almost the same time as the BL case and there were no signs of the flashover delay observed in the experiment. The igniters HRR-Time input curves were then shifted by 150 s (same difference in time to flashover between D1 and the BL case experimentally). It was then found that the HRR-time curve matched with the experimental curve of the DD experiment. Therefore, the delay in time to flashover in D1 compared to the BL in the experiments is probably due to a human error in igniting the wood cribs in D1.

Additionally, significant differences between this experiment and other experiments in literature were found when comparing the results to the work done in [48] which was of similar design and fuel load of this work (i.e., DD experiment) with two IS dwellings at 1.0 m away in an opening to wall orientation. The flashover of D1 in [48] was found to be around 200 s (e.g., ≈− 60% compared to D1 in the DD experiment). Therefore, currently, there is no evidence in the literature that placing D2 at 1.0 m from D1 can influence the pre-flashover phase of D1. In the rest of this modelling section, the shifted igniters were used to better represent the fire dynamics in the experiments and replicate it.

The “New Igniters” model shows a good agreement with the experimental results in terms of presenting a minor difference between the values and characterizing the overall fire behavior of the double dwelling fire experiment. The crucial distinction between the “New Igniters” design and the “Old Igniters” model lies in the ignition method. The “New Igniters” model employs igniters that are ramped over a period of time, resulting in a delay of approximately 150 seconds compared to the igniters utilized in the “Old Igniters” model. In respect of time to flashover, the “Old Igniters” model is highly representative to the single BL case where both of them to reach flashover with 150 s in advance compared to the “New Igniters” model and the experimental outcome.

**Figure 6:**
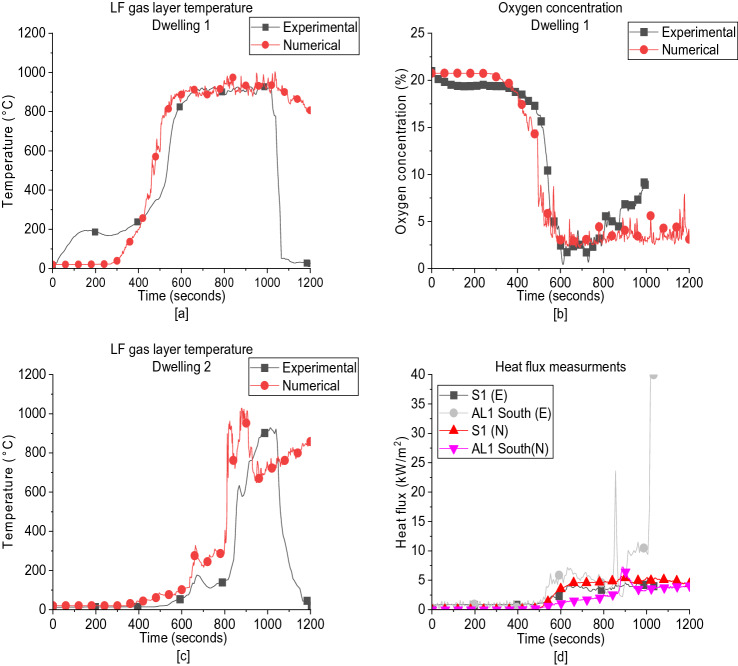
(**a**) Gas layer temperature at the left front of dwelling 1; (**b**) Oxygen concentration at Dwelling 1; (**c**) Gas layer temperature at the left front of Dwelling 2; (**d**) Heat flux measurements at the South alley (AL 1) and at the North wall of Dwelling 1 (S1), where E is experimental, and N is numerical

As presented in Figure 6a based on the shifted igniters, FDS managed to capture the fire dynamics in D1, where the top Left Front (LF) gas layer temperature was accurately replicated via FDS post-flashover. The oxygen concentration at the back of the compartment was also captured by FDS with identical behaviour and values post-flashover as presented in Figure 6b. As presented in Figure 6c, FDS model overestimated the LF gas layer temperature in post-flashover ignition of the cardboard and underestimated it post-flashover of D2’s wood cribs with average variation of 15% to 20%. FDS captured the same values and behaviour for the side heat fluxes from D1 post-flashover. It, however, underestimated the heat fluxes at the alley (between the two dwellings). The thermal radiation from the flames can be affected by the cell size, the number of radiation angles and even the usage of shrink factor. It is recommended to replicate the same model with smaller cell sizes and more radiation angles to get better estimations of the heat fluxes in the alley. However, this model still gives very good indication to all the other aspects of this experiment (e.g., HRR-time curves, fire spread timeline, gas layer profiles and combustion efficiency within the compartment). The comparison of the heat flux measurements at the South alley (AL 1) and at the North wall of Dwelling 1 (S1) is given in Figure 6d. For S1, FDS shows a considerable high performance in depicting the experimental heat flux values, while for AL1 South, FDS significantly underestimates the values of the experiment and even fails to catch the qualitative behavior.

In general, the numerical simulations reproduced the main fire dynamics and trends within the double ISD. The HRR was reproduced numerically with very high accuracy in respect of experimental results. The gas layer temperature at the left front of both dwellings shows a very good agreement with data from experiment which emphasize on the high numerical efficiency of the model used. The oxygen concentration was well captured at D1. The incident heat flux at the North wall of D1 was well replicated, while incident heat flux at South alley was quantitatively underestimated by the model despite of keeping the same qualitative behavior.

### Parametric Study

The effect of wind on the fire dynamics of the dwelling of origin was studied earlier in this section. Therefore, the fire in D1 was accelerated to reach flashover earlier to avoid lengthy simulations and only focus on the fire spread mechanism to D2 and the development of the fire dynamics in D2 post-ignition. To accelerate the fire development in D1, each wood crib was assumed to start ignition from time zero. In this section, for D2 the flashover criteria was based on the sudden drop in the oxygen concentration measurements within the compartment.

The numerical results are split into 4 sections as the following:

- Sect. 4.2.1. Ignition of D2;
- Sect. 4.2.2. Flashover of D2;
- Sect. 4.2.3. Heat transfer analysis;
- Sect. 4.2.4. Heat fluxes to the surroundings.

#### Ignition of D2

The cardboard ignition time (t_ig_) for each case is shown in Table 1, where t_ig_ is the time from the start of ignition in D1 (time = 0.0 s) till the cardboard ignition in D2. The results show that for all wind speeds where D2’s front wall was windward (E) or leeward (W) the cardboard ignited much earlier than when the wind direction was blowing in the alley (N/S) (i.e., parallel to the front wall). These observations are also consistent with the net heat energy (E_net_) average values (e.g., from time 0 to cardboard ignition for each case). Where, the net heat energy is the energy measured via a vertical slice file at 0.2 m from D2’s front wall (outside). Where negative value means more energy is flowing from D1 to D2 and vice versa. The highest E_net_ was measured for the DD case (i.e., double dwelling with no wind), which demonstrates that the induced wind will always decrease the total energy present within the alley between the two dwellings. This observation also reflected clearly on the time to ignition, where the DD case experienced the fastest spread between all the other cases (except for the 10 E/W, which will be discussed later). The E_net_, therefore, presents a new concept that can be used in future studies to estimate the potential of fire spread in different conditions.

**Table 1:** Ignition of Dwelling 2 at Different Wind Conditions, the Net Energy Between the Two Dwelling and Time to Flashover of Dwelling 2

Case	DD	6E	6S	6W	6N	10E	10S	10W	10N	14E	14S	14W	14N
t_ig_ (s)	28	32	48	32	78	24	58	22	115	46	60	38	128
%		+ 14	+ 71	+ 14	+ 178	− 14	+ 107	− 21	+ 310	+ 64	+ 114	+ 35	+ 357
E_net_ (kW)	− 484	− 291	− 61	− 128	− 10	− 276	− 47	− 163	0	− 452	− 17	− 166	5
%		− 40	− 87	− 74	− 98	+ 43	− 90	− 66	–	− 7	− 96	− 66	–
t_fo_ (s)	326	248	290	316	362	198	354	226	–	112	358	140	–
%		− 24	− 11	− 3	+ 11	− 39	+ 8	− 31	–	− 66	+ 10	− 57	–

*Note:* Each value was presented in terms of percentage in comparison to DD (baseline) scenario

**Figure 7:**
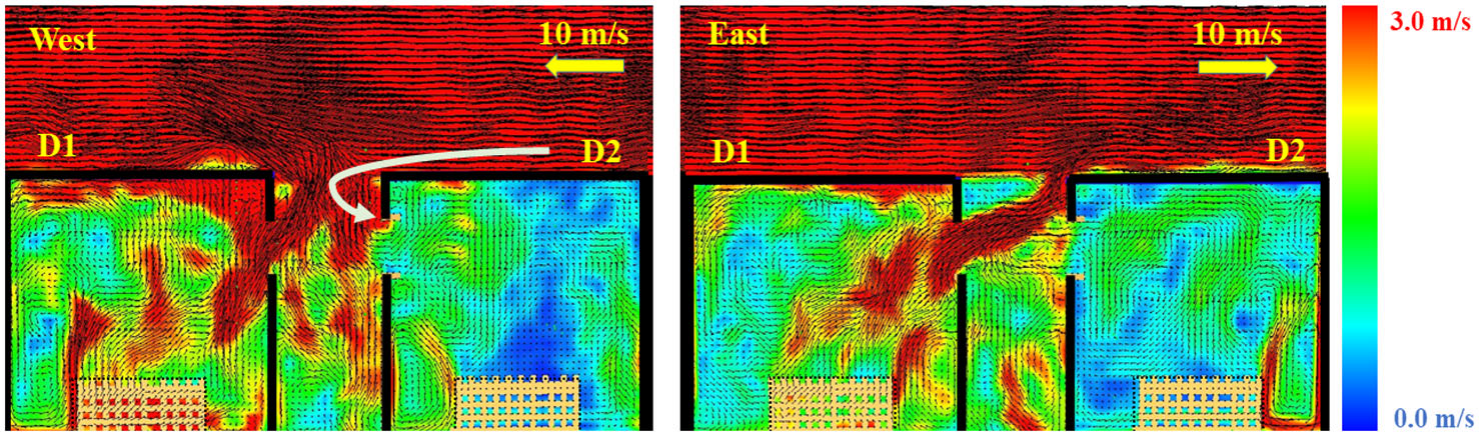
Velocity vector slice file for cases 10W and 10E averaged from time 0 to cardboard ignition in D2

As expected, as the wind speed in the N and S directions increases, the E_net_ and the potential of the fire spread decreases. Therefore, the E_net_ managed to replicate the fire spread potential trends for the N and S cases and also differentiated between the N/S and E/W cases. However, that was not the case when comparing the E and W cases. It was found that the E_net_ values are generally higher for the E cases, while the W cases showed quicker fire spread (lower t_ig_). That was not expected as the W case is an opposed fire spread case, where the fire is spreading against the wind direction. To further understand this, a velocity slice file at the centre of the window of each case was developed and averaged over time from time = 0.0 to t_ig_ for each case, as presented in Figure 7.

Figure 7 clearly shows that for the W case, the wind is tilting (pushing) the external plume from D1 away from D2, while for case E, the wind is enhancing the flame impingement from D1 on D2. Looking from only this perspective, one can expect a much faster fire spread from the E case compared to the W case. However, as the wind streams from the W direction encounters a sudden change in the flow path at the front edge of the roof of D2 and generates a recirculating zone at the edge of D2 roof. This can be explained by a fluid and aerodynamics phenomenon known as flow detachment, back-ward facing step or flow separation [49]. With high-speed flow passing over the surface (e.g., roof) then experiencing a sudden change in the flow path (e.g., end of the roof surface) turbulent boundary layer will cause a flow separation which will re-attach downstream of D2 and cause recirculating zone in the region between the edge of D2 and the external plume from D1, due to the pressure drop. The pressure drop produced will then drag a portion of the external plume from D1 (which falls within the recirculating zone) to flow towards D2 and cause ignition. This observation or phenomena is unlikely to be seen if the distance between the two dwelling and the external plume from D1 was bigger and hence would be totally out of the recirculating zone. This cannot be assumed to happen in most real cases as the wind flow will very rarely be as smooth as it is modelled in this study. It is, therefore, recommended to study the wind in both lab-based conditions and outdoors conditions to further understand the probability of this phenomenon to have a significant effect in real scenarios.

It is always expected that the external plume from D1 would be pushed (tilted) towards D2 and the plume impingement would be enhanced as the wind speed increases. It is important also to re-call the observations from Lemmertz et al. [19], which support that as the wind speed increases on the back wall of D1 the flashover will occur relatively faster. This effect should be taken into consideration when studying the fire spread severity between two dwellings in wind conditions. It was observed in Lemmertz et al. [19] that the effect of the wind speed on the time to flashover in the fire ISD was significant for thermally-thin bounded compartment when the wind increased from 5  m/s to 10 m/s, post 10 m/s the wind effect on the time to flashover was negligible. Additionally Lemmertz et al. observed that radiative heat fluxes infront of the dwelling’s opening increased with the wind speed. This observation matches the findings from Rush et al. [44] where the 10 m/s wind speed was also found to coincide with the peak heat fluxes from the openings of the dwelling of origin to the surroundings. These observations will help in understanding/discussing the ${t}_{ig}$ for E cases.

The moment of ignition (${t}_{ig}$) of dwelling 2 relies on the times of:

- D1 reaching flashover,
- the external plume is fully developed, and,
- cardboard ignites.

**Figure 8:**
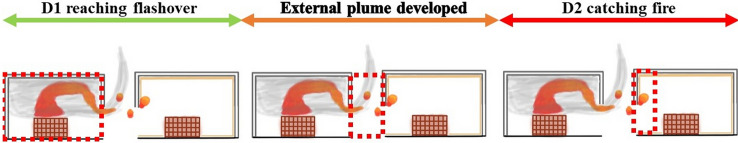
Simple schematic for the fire spread via flame impingement and radiation

This is presented with illustration in a simple schematic in Figure 8.

##### Comparing 6E and 10E

As presented in Table 1, D2 ignited earlier in 10E (*t*_*ig*_ = 24 s) compared to 6E (*t*_*ig*_ = 32 s). In [19] it was observed that the time to flashover for a single dwelling significantly decreased when wind increased from 5 to 10 m/s, which means that D1 is expected to reach flashover much earlier for case 10 m/s compared to 6 m/s (shorter time for the green zone in Figure 8). Additionally, in [19] it was found that when the wind speed increased from 5 to 10 m/s the external plume developed faster (i.e., Global Equivalence Ratio (GER) > 1 is reached earlier) for a single dwelling. This matched the observation in this study where the external plume for the 10E case developed (orange zone in Figure 8) earlier compared to the 6E case. Based on that, comparing cases 6E and 10E, it was found that as wind speed increases, ${t}_{ig}$ will decrease.

##### Comparing 6E and 14E

As presented in Table 1, the ignition time, ${t}_{ig}$ (the time from the start of ignition in D1 till the cardboard ignition in D2) for 6E case was 32 s, while 14E was 42 s (i.e., time increased by + 32%). Based on the study conducted in Lemmertz et al. [19], as the wind speed increased from 5 m/s to 15 m/s, the time for the single dwelling to reach flashover decreased, additionally, the (GER) > 1 condition was also reached earlier. Therefore, the result in this study contradicts the expectations from earlier studies that the ignition of D2 will occur earlier with 14E case compared to the 6E case. This can be explained as for higher wind speed (in this configuration), there was enough air entrainment inside the compartment and less unburnt gases were venting post-flashover and hence longer time to reach a fully developed external plume. Hence, while the GER calculations at the door seem to give a good indication for the time to flashover, it might not be reflecting the condition of the sustained external plume post-flashover in this study.

**Figure 9:**
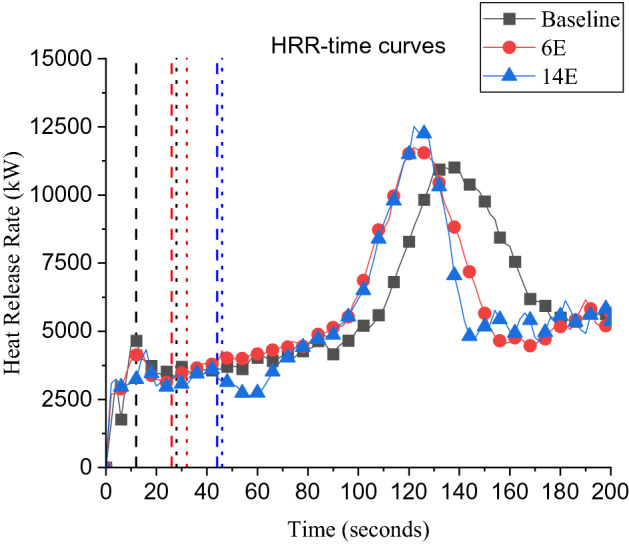
Time-line for the plume ejection from D1 and the cardboard ignition in D2 (where the dashed lines: time when sustained external plume from D1 was captured, dotted lines: time when cardboard ignited for each case), BL refers to the results from the benchmarked model of the two dwellings fire spread experiment with no wind

For this study, these observations were further investigated as in Figure 9, which shows the HRR-time curves with the flashover time for cases DD, 6E and 14E. It was found that, the external plume from case 6E was observed at around 25 s (and it took around 7 s to 8 s for D2 to ignite. However, for 14E case the external plume was observed at 45 s (around 100% longer time compared to 6E) and it took only 1 s for D2 to ignite (red zone in Figure 8). This also shows the effect of the wind speed on the ignition of D2 once the external plume is fully developed to be very quick with high wind speeds. The effect of the S and N directions was clearly different than that of E and W. The wind speed in the S and N directions was directly proportional to the ${\text{t}}_{\text{ig}}$. These wind directions (i.e., S and N) have the feature to move in the alley between D1 and D2. Consequently, the wind flow will get through the openings of both dwellings and will significantly affect the combustion process, overall fire dynamics and the external plume from D1. Additionally, for cases S and N the E_net_ was relatively lower compared to the E and W cases, which was expected due to the fact that in these directions (S and N) the wind will tilt the external plume sideward away from D2. Therefore, generally, S and N cases led to longer times for the fire to spread from D1 to D2.

##### Comparing S and N

It was found that N cases took longer time for the fire to spread to D2 than S cases. The only difference between these two cases is the wind direction relative to the door and window openings. For the S cases, the wind meets the door first, therefore, most of the wind flow just enters the compartment (similar to the normal cold flow entering a compartment fire). This flow increases the pressure within the compartment and pushes most of the unburned gases within the compartment externally and hence (due to the high forced flow within D1) the external plume appears and sustains earlier for case S compared to N case.

For the N cases, the wind flow meets the window first and hence it conducts the opposite effect to the external plume from the window, it pushes it back to the inside of the compartment. Therefore, for the N case the cold fresh air is entering the compartment from both the door and the window. Hence the N cases experienced the longest fire spread time (${t}_{ig}$).

**Figure 10:**
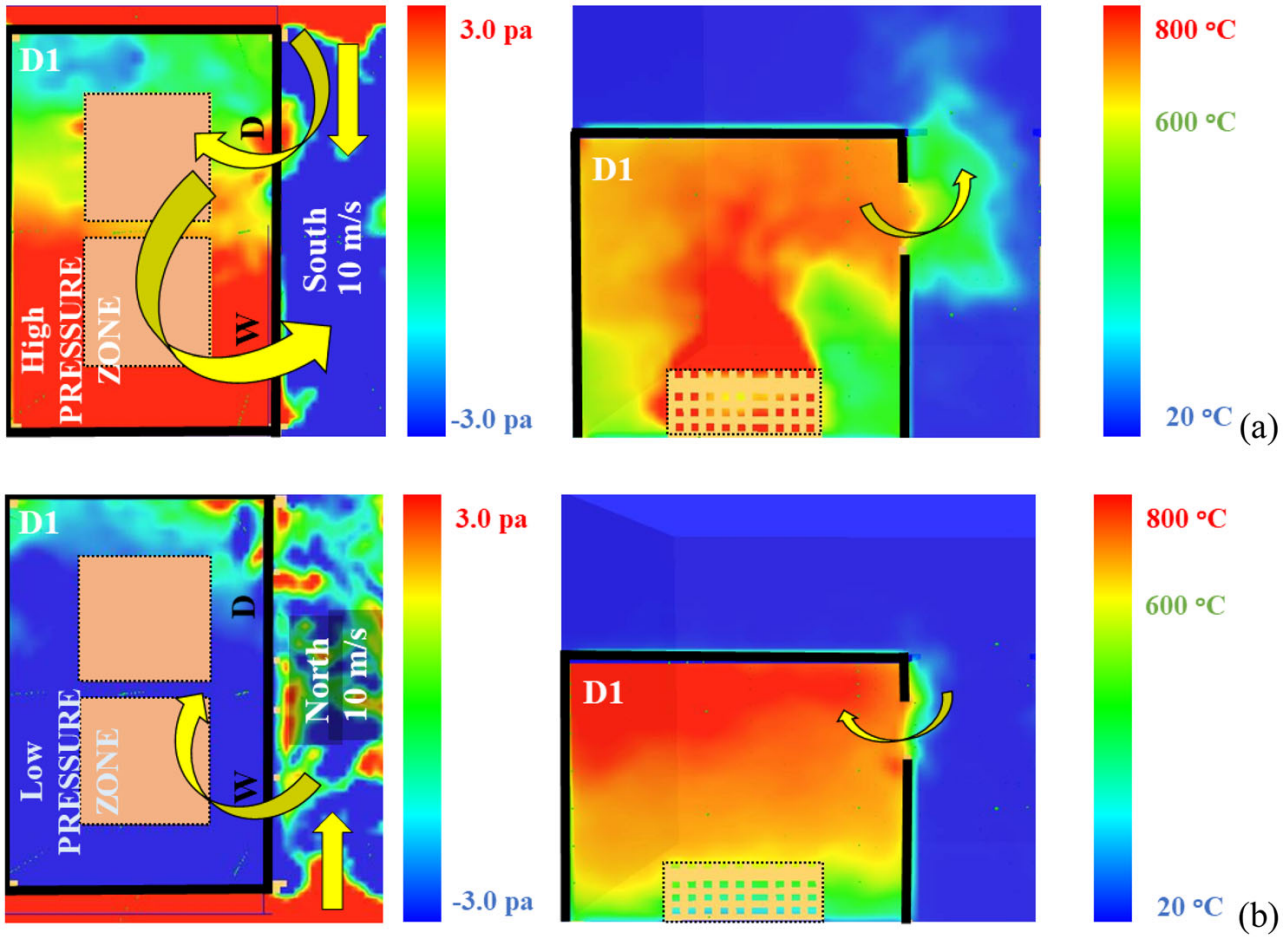
Horizontal (upper view) pressure slice and vertical (side view) temperature slice files averaged from time zero to the time of ignition for cases (**a**) 10S and (**b**) 10N

**Figure 11:**
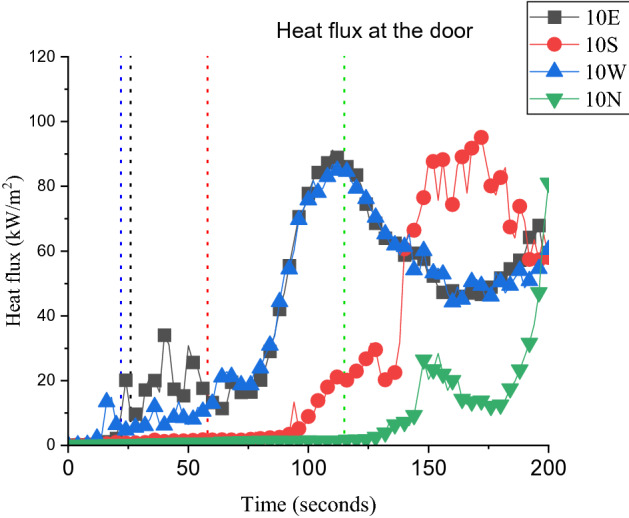
The heat flux measured at the top edge of the door of D2 (where the dotted lines represent the cardboard ignition for each case)

To explain this behaviour, Figure 10 shows horizontal pressure slice (at Z = 1.5 m) and vertical temperature slice (at the middle of the window) files averaged from the time zero to the ${t}_{ig}$ for cases (a) 10S and (b) 10N. Based on Figure 10a, it is clear that wind entering through the door increased the pressure within the compartment (specially the area at the back of the window) and that reflected on the temperature slice, which shows the hot gases being pushed out of the compartment. On the contrary, Figure 10b presents a low-pressure zone within the whole dwelling and significantly less external hot gases jet from the window. Looking at the measured heat fluxes at the top edge of the door of D2 as presented in Figure 11. It is shown that the E and W cases experienced a sudden increase in the measured heat fluxes which matches the ${t}_{ig}$ of each case. This is believed to be due to the direct flame impingement from D1 on D2. However, Cases N and S showed very low heat flux values even after the ${t}_{ig}$. This is expected, as the external plume was tilted away from D2 and reduced the possibility of flame impingement. Therefore, the fire spread mechanism for these cases can be due to the accumulated heat fluxes on the cells around or on the cardboard surface and hence reaching the ignition temperature of the cardboard (probably at the window first rather than the door). Generally, it was found that the openings’ location with respect to the wind direction will have a significant effect on the ignition of D2. Based on the conditions in this study, the different cases were ranked based on the risk of fire spread as the following; (1) W where the front wall of D2 is leeward; (2) E where the front of D2 is windward; (3) S where the front wall of D2 is sideward with wind stream reaching the door opening first; and, (4) N where the front wall of D2 is sideward with wind stream reaching the window opening first.

#### Dwelling 2 Flashover

##### Windward (E) and Leeward (W) Cases

**Figure 12:**
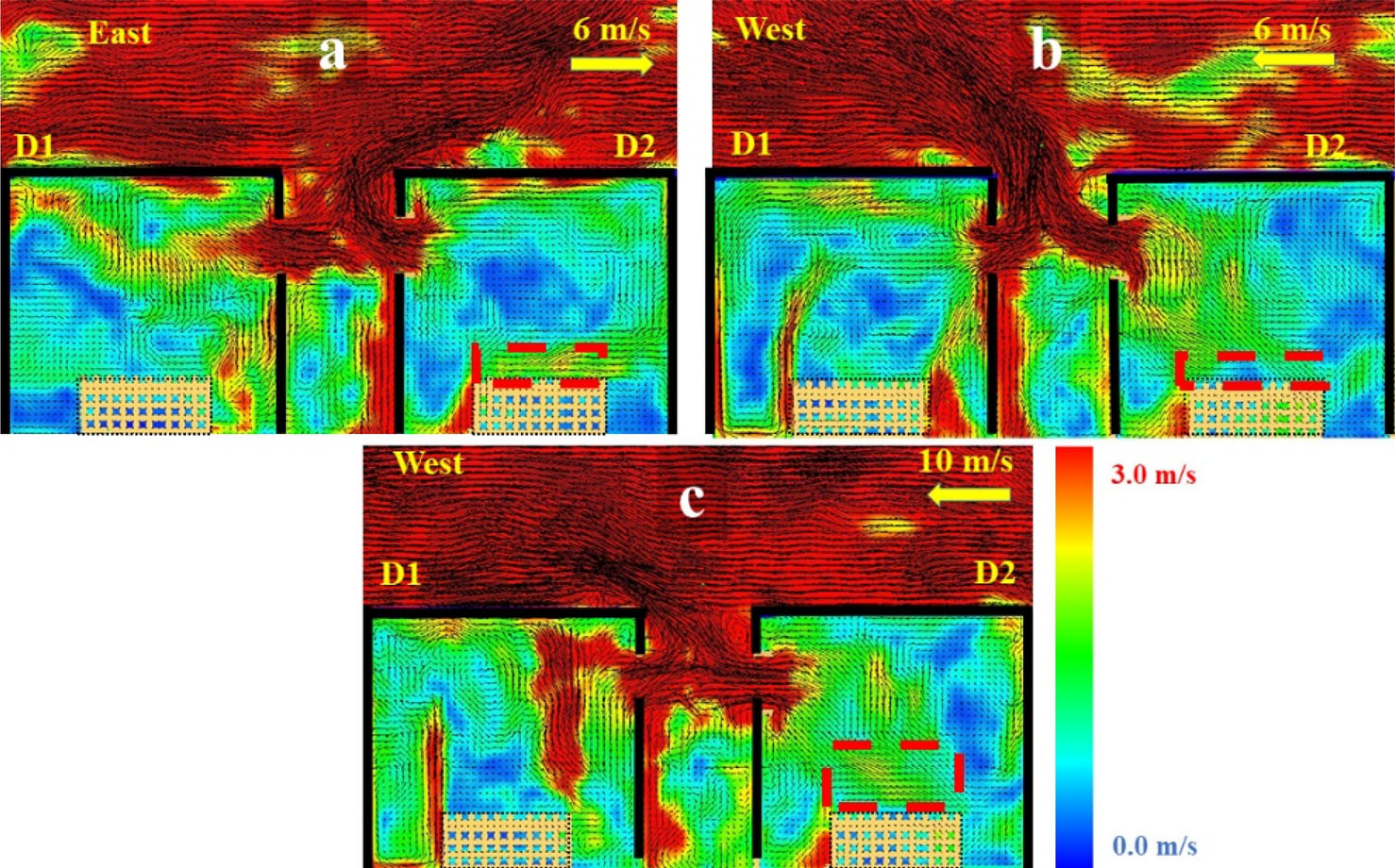
Vertical averaged velocity slice file at the window of cases (**a**) 6E, (**b**) 6W and (**c**) 10W averaged from the time of cardboard ignition to flashover for each case

For cases where the front wall of D2 is windward (E) or leeward (W), the ${t}_{fo}$ is inversely proportional to the wind speed. Increasing the wind speed was found to enhance the mixing efficiency within the compartment by decreasing the mixing timescale leading to faster time to reach the flashover criteria, this was also observed for the leeward case in Lemmertz et al. [19]. Comparing the E and W cases for each wind speed, it was found that for the windward cases (E), the wind flow will have higher effect on enhancing the mixing within the compartment compared to the leeward cases (W). Figure 12 presents the averaged velocity field profile within D2 averaged between the time of cardboard ignition and flashover in D2. It was observed with the aid of Figure 12a, b that for the same wind speed (i.e., 6E and 6W), the induced air flow from the wind in the 6E case was more uniform compared to 6W and there is clearly better air entrainment around the wood crib surface (in case 6E). This is highlighted via red dashed boxes on Figure 12a, b, (higher velocities, represented by green colour around the crib). Therefore, the ${t}_{fo}$ was 248 s and 316 s for cases 6E and 6W, respectively. However, comparing 6E with 10W (Figure 12a–c), it was found that there is higher entrainment around the wood crib surface for case 10W. Therefore, the ${t}_{fo}$ was 248 s and 226 s for cases 6E and 10W, respectively. From these observations it can be concluded that the time to flashover (for lee and windward cases) is both direction and speed dependant, i.e., for same wind speed E cases will have better entrainment than W cases, yet, for low E speeds and high W speeds, the W cases will have better entrainment.

##### Side Walls Wind (S and N)

**Figure 13:**
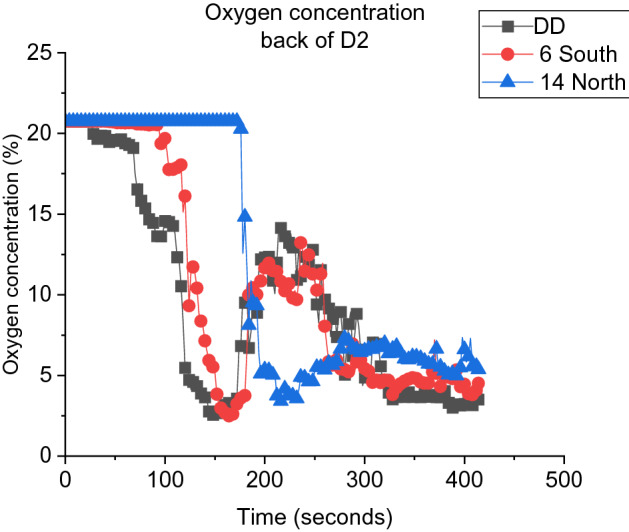
Oxygen concentration at the back of D2 for BL, 6S and 14N

**Figure 14:**
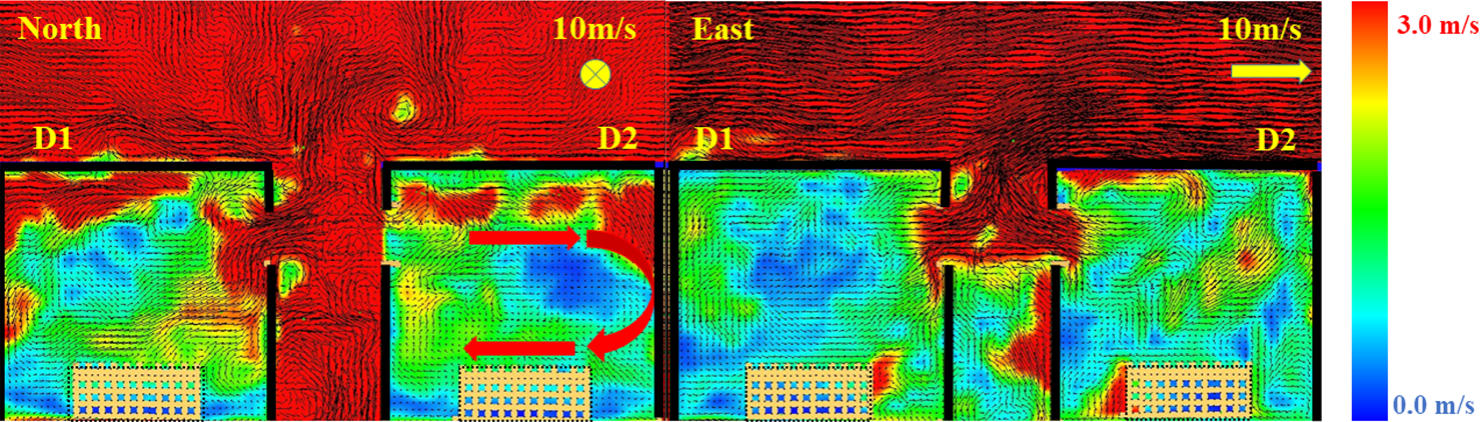
Vertical averaged velocity slice file at the window of cases 10N and 10E, averaged for 200 s from cardboard extinguishment for each case

Contrary to E and W, the wind speed is proportional to the time to flashover for cases S and N. The wind effect in D2 when the wind was blowing from the E and W was mainly increasing the mixing due to the nature of these directions (i.e., flow direction is perpendicular to the openings plan). However, for N and S cases (i.e., flow parallel to the openings plan), the induced wind cools down the compartment, especially for the N cases with high wind speeds (i.e., 10 m/s and 14 m/s). Similar to what was observed in the cardboard ignition, the N case (the case that reaches the window opening first) showed the lowest potential of reaching flashover in D2. Where 10-14N cases did not reach flashover for D2 after the ignition of the cardboard and 6N case showed the highest ${t}_{fo}$. Note: it was defined as a no-flashover case if there was no external plume presented observed and the oxygen concentration at the back did not drop to the expected limit (i.e., for 10 min from the flashover of D1) This is shown in Figure 13, where 14N case is compared to the no wind (DD, baseline case) and 6S cases. To further investigate the velocity field around the wood crib’s surface, Figure 14 shows the velocity field around the crib’s surface for cases 10N and 10E. This figure highlights the high velocities field (i.e., green and red fields) around the wood crib’s surface for the 10N compared to 10E, where 10N did not reach flashover. This is expected to be due to the high wind speed below the celling (i.e., red fields) cooling the gas layer and also the flow field over the crib’s surface (shown with red arrows) where it cools down the surface and also entrains more air within the crib. However, due to the flow field of the fresh air, the speed and the cooling of the gas layer, the cooling effect dominates the entrainment effect for the N cases with high wind speeds. Yet at lower wind speed (i.e., 6 m/s) the air entrainment effect dominates. Generally, at medium and high wind speeds (i.e., 10 m/s and 14 m/s) S and N cases will take significantly longer time to reach flashover in D2 compared to E and W. That is due to the cooling effect for both the gas layer and the surface of the crib.

##### Upper Gas Layer Temperature at t_fo_

**Figure 15:**
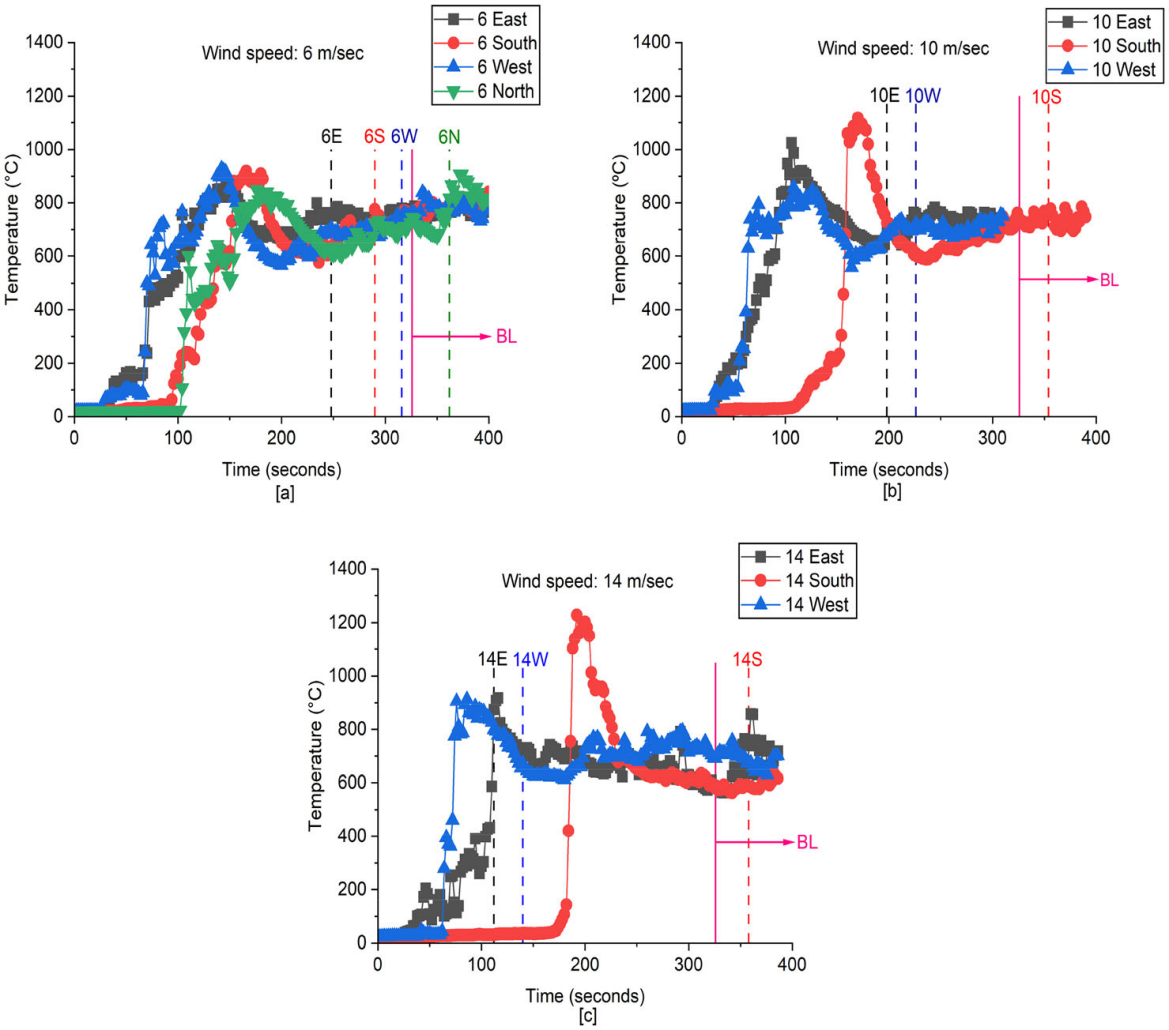
The gas layer temperature near the ceiling (TC_10) at the left back of D2. Dashed lines correspond to t_**fo**_ at each case associated with wind and the purple solid line is used to show t_**fo**_ of BL fire scenario. (**a**) 6 m/s, (**b**) 10 m/s, and (**c**) 14 m/s

**6 m/s:** Figure 15a indicates that the upper gas layer temperature in D2 has the same trends for all wind directions. The time required to reach flashover for 6E, 6S and 6W was 250 s, 290 s, and 315 s, respectively. For 6N, the time to flashover was longer (i.e., 360 s) and coupled with upper gas layer temperature (i.e., 815 °C), which suggests that more energy and time is needed within D2 to obtain a hot upper gas layer temperature sufficient for flashover because of the highly high cooling effect associated with this wind direction.

**10 m/s:** As shown in Figure 15b, the temperature of the gas layer in D2 has the same trends for all wind directions, however it takes substantially longer time for 10S and 10N to generate high upper gas layer temperatures. This indicates that the cooling effect of wind in these specific directions (South and North) rises as its velocity increases. As a result, the time required in D2 with 10S to reach flashover increased to 355 s, whilst with 10N it did not reach flashover. Clearly, 10S requires more time than 10E and 10W for heat to accumulate and reach high gas temperatures sufficient for flashover. Additionally, the time to attain flashover in D2 with 10E is quicker than with 10W, which may be attributed to the orientation of D2's openings towards 10E, resulting in an enhanced rate of air entrainment compared to 10W.

**14 m/s:** Figure 15c demonstrates the same behaviour for the hot gas layer when the wind speed increased to 14 m/s as it does when the wind speed is 10 m/s, but with a significant delay in building up a high upper gas layer temperature for 14S and 14N due to the extremely high cooling effect of wind in these specific directions (S and N), as the wind speed increases. As a result, the time required to reach flashover for 14S increased to 360 s, whilst no flashover recorded for 14N. At the moment of flashover, there was a significant difference in the temperature of the hot gas layer between 14E (870 °C) and 14W (670 °C) because wind blowing to the W direction wind has a tendency to cool the back of D2 at high wind speeds, but wind blowing to the E direction improves the combustion efficiency within D2.

For directions E, S, and W, the time to reach flashover is shorter than that for the BL case and the gas layer temperature at time of flashover is between 690 °C and 775 °C at wind speeds of 6 m/s and 10 m/s, which is lower than the BL (795 °C). This indicates that the air flow accompanied by wind speed 6 m/s and 10 m/s, is anticipated to reduce the gas layer temperature at flashover at all directions, and to enhance the burning rate except for 10S which has flashover at 355 s longer than the BL (325 s). As the wind speed increased to14 m/s, the upper hot gas layer of 14E at flashover time acquired a higher value (875 °C) at shorter time (110 s) because of the increased burning rate and better mixing between the pyrolyzed gases and fresh air, while the hot gas layer of 14W at flashover time (670 °C) is getting lower due to the increased cooling rate from the back of D2. Nevertheless, it is clear that the wood cribs burning rate is better at high wind speed (14 m/s) which can be observed from the decreased flashover time (14E and 14W, 112 s and 140 s, respectively) compared to the BL. For 14S, it can be interpreted that the elevated cooling rate due to the entrained air is dominating over its ability to improve the heating rate and the combustion efficiency inside D2 resulting in gas layer temperature of 590 °C and flashover time of 360 s. On the other side, 10N and 14N didn’t reach flashover and hence their values are not shown in Figure 15.

#### Heat Transfer Analysis

##### Overall Heat Transfer Coefficient (U)

**Figure 16:**
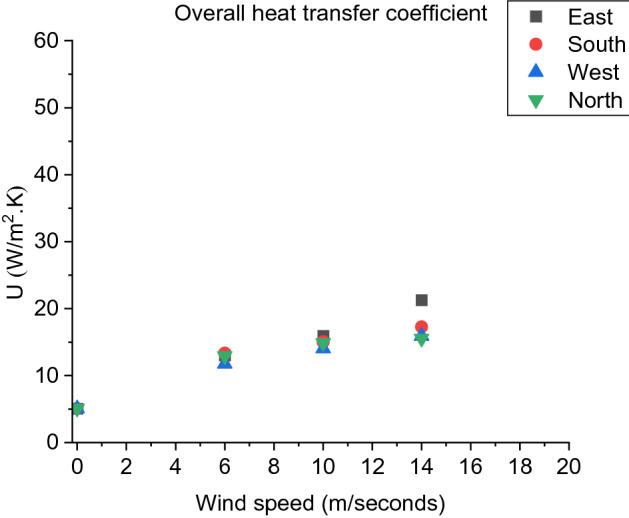
The overall heat transfer coefficient variation with wind speed for each wind direction

Heat transfer analysis is conducted in this section based on the theoretical heat transfer analysis methodology presented in Sect. 3.3. As shown in Figure 16, the overall heat transfer coefficient (U) is directly proportional to wind speed for each wind direction, indicating that the interaction between the surronding gases and the dwelling boundaries (D2) increases as the wind speed increases, resulting in higher rate of heat transfer. Where the results were averaged from the time of ignition of D2 up till the end of the simulation.

**Figure 17:**
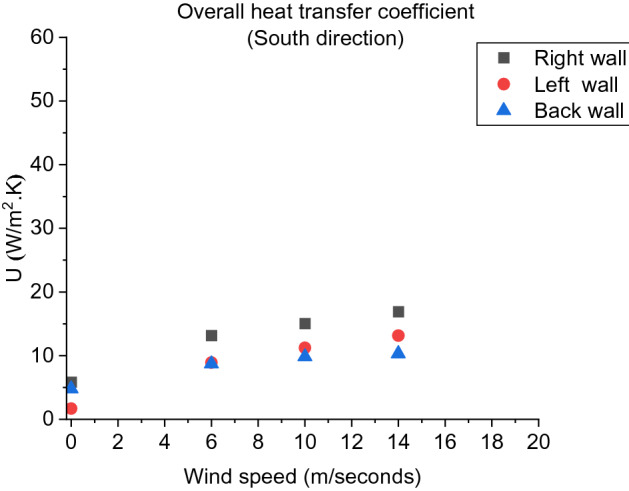
The overall heat transfer coefficient (U) variation with wind speed for South direction at each wall

As shown in Figure 17, it was also observed that, regardless the wind direction and speed, the right wall (next to the door) of D2 is experiencing the highest total heat transfer ($U$) among all scenarios in this study. This is expected to be due to the location of this wall being next to the largest opening factor (door). Therefore, in respect of heat transfer losses to and from boundaries, the ventilation opening-wall distance is commonly dominating over the effect of wind conditions for each particular case. In other words, the opening ventilation factor in addition to its spatial location and distance from the walls are playing an important role when analysing the heat transfer of a dwelling.

##### Radiative Heat Transfer Coefficient

**Figure 18:**
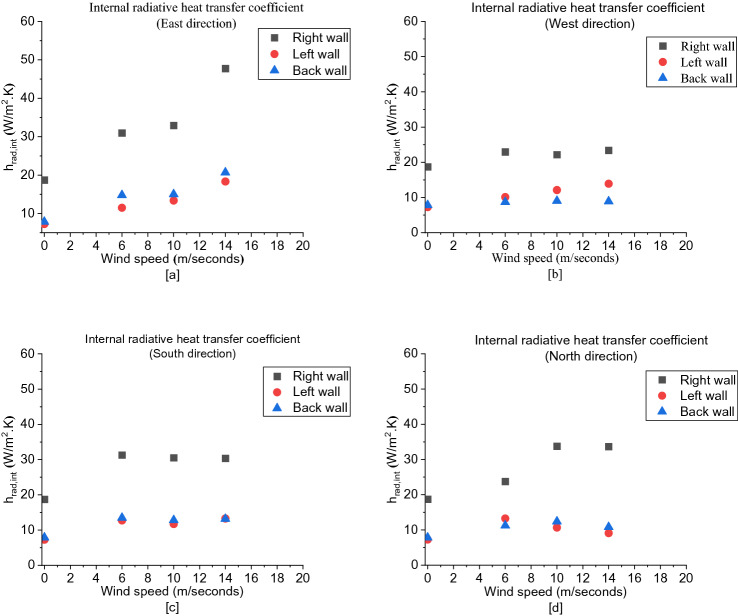
The internal radiative heat transfer coefficient (h_rad_,_int_) for: (**a**) East, (**b**) West, (**c**) South and (**d**) North cases

The influence of wind on the internal radiative heat transfer coefficient (*h*_*rad,in*_) is approximately the same as on $U$, as shown in Figure 18. Additionally, it was found that the right wall had the highest *h*_*rad,in*_ in each case compared to the left and back walls which had approximately very close values for most cases.

**Figure 19:**
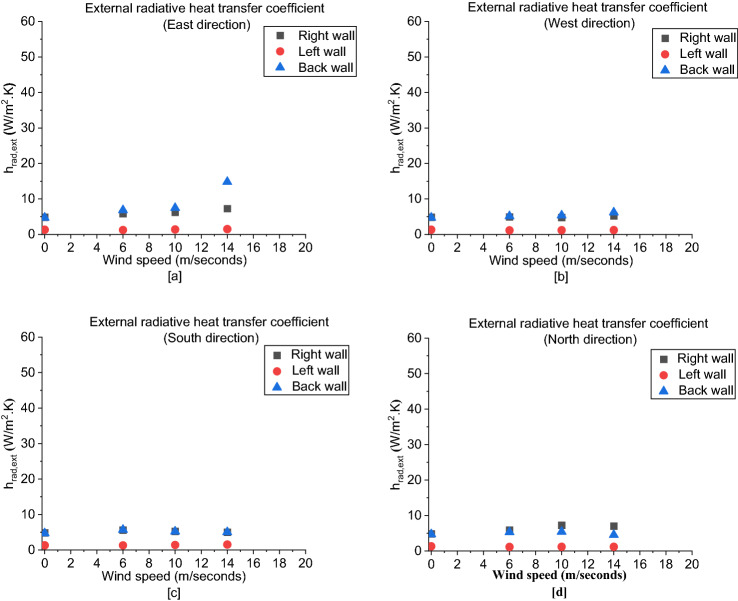
The external radiative heat transfer coefficient (h_rad_,_ext_) for: (**a**) East, (**b**) West, (**c**) Soth and (**d**) North cases

**Figure 20:**
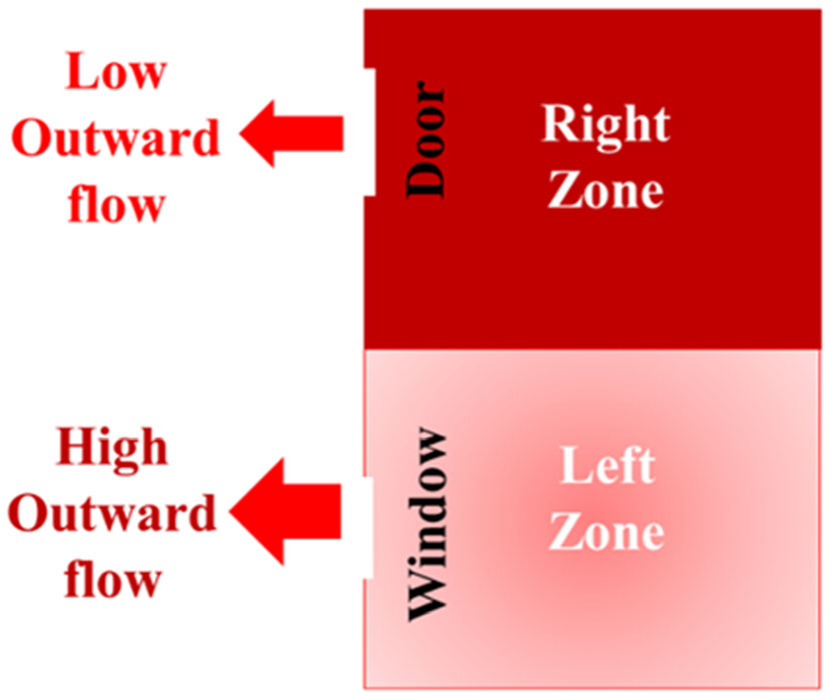
Plan view showing zone 1 (right zone) and zone 2 (left zone) for D2

For the external radiative heat transfer coefficient (*h*_*rad,ext*_), as presented in Figure 19, the left walls are having the lowest *h*_*rad,ext*_. To investigate the reason behind this observation, D2 is divided into two zones as shown Figure 20, where the right and left zones are where the door and window are located, respectively. It was found that the mass outflow and flow velocities at the window are higher than that at the door, hence more hot gas are leaving the left zone. The right zone is always well-entrained through the cold fresh air entering at the door and hence higher combustion efficiency and flow exchange occurring within this zone. The higher the combustion efficiency led to higher overall wall and gases temperatures within this zone and hence higher radiative heat transfer coefficients (*h*_*rad*_).

##### Convective Heat Transfer Coefficient

**Figure 21:**
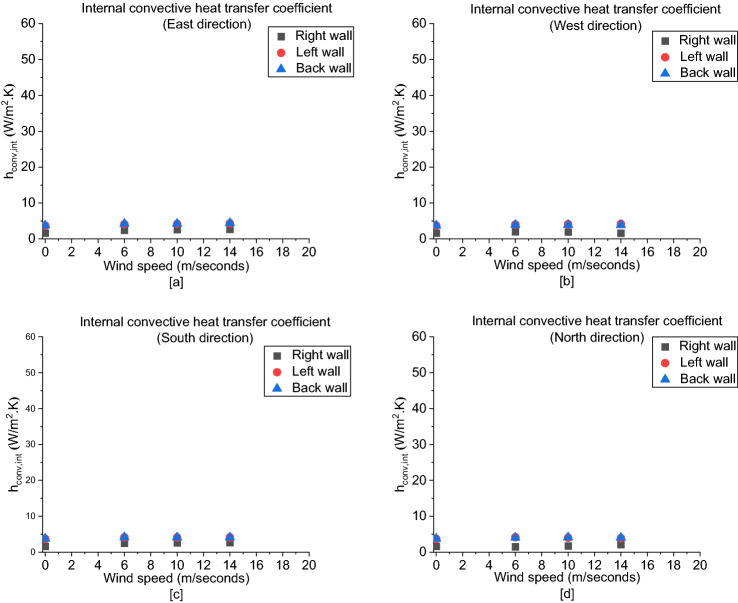
The internal convective heat transfer coefficient (h_conv_,_int_)

**Figure 22:**
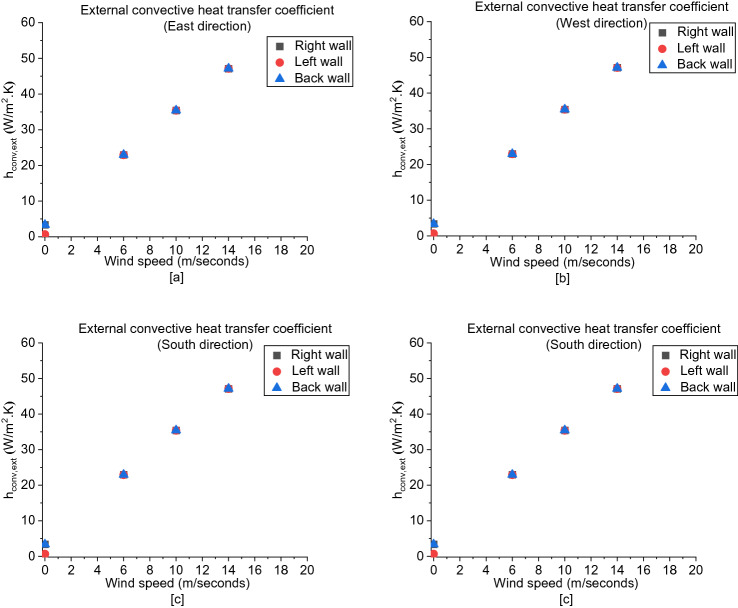
The external convective heat transfer coefficient (h_conv_,_ext_)

Regarding the internal convective heat transfer coefficient (h_conv_,_int_), Figure 21 shows the values at the left wall and back wall are approximately the same with very small variations whereas the values at the right wall are around half the values at the other walls with a negligible change for all cases, which can be due to the less heat transfer in the right zone leading to have a higher gas and wall temperatures with a very small difference. On the other hand, as shown in Figure 22, the external convective heat transfer coefficient (h_conv_,_ext_) is almost not varying with respect to different walls and wind directions. However, it only increases by increasing the wind speed as a result of the forced convection correlation is only a function of wind velocity and doesn’t account to wind direction. All in all, it is determined that the overall heat transfer coefficient (U) is significantly affected qualitatively by the internal radiative heat transfer coefficient (h_rad_,_int_) which shows a remarkable variation with wind speed and direction with respect to each wall.

#### Heat Flux at Each Alley

The heat fluxes were compared for all cases at the north and south of the alley to further understand the potential fire spread risk to adjacent dwellings for different wind speeds.

##### South Region of the Alley Between Dwellings

**Figure 23:**
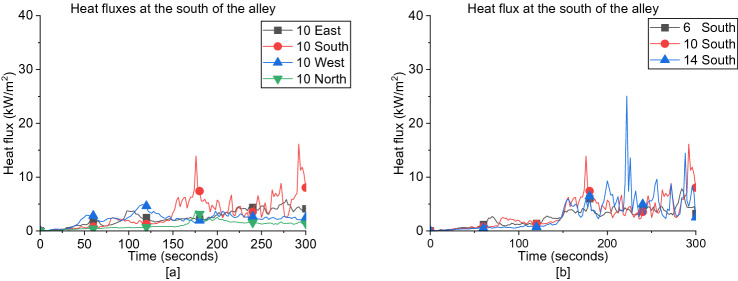
Heat fluxes at the south of the alley for (**a**) all wind directions at 10 m/s and (**b**) South wind cases for different wind speeds

As presented in Figure 23a, for the south region of the alley, the incident heat flux of cases subjected to S wind direction is higher than all other directions for each wind speed and approximately no variation on average with the increase of wind speed as shown in Figure 23b. For the opposed wind directions (i.e., E vs. W and N vs. S), the heat flux of E and W wind direction is approximately similar to each other, while the heat flux of N wind direction is always lower compared to the S direction.

##### North Region of the Alley Between Dwellings

**Figure 24:**
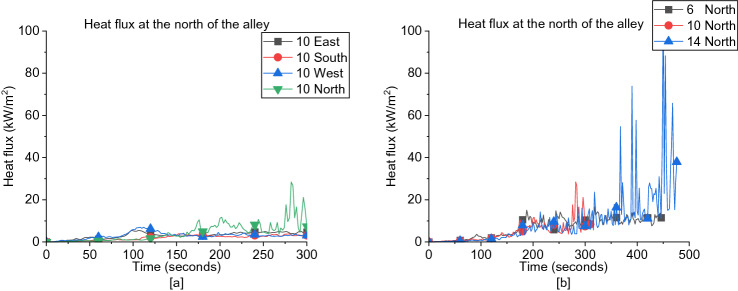
Heat fluxes at the north of the alley for (**a**) all wind directions at 10 m/s and (**b**) N wind direction cases for different wind speeds

In contrast, the incident heat flux at the North region of the alley is having higher values when exposed to N wind direction than other directions for each wind speed as illustrated in Figure 24a, which shows approximately no variation with the increase of wind speed as given in Figure 24b. Same behaviour is observed as of the North alley where for each certain speed, the heat flux of E and W wind directions is approximately similar to each other with a slight decrease as the wind speed increases while the heat flux of S wind direction is always lower. For both regions of the alley, it is shown that varying the wind speed from 6 m/s to 14 m/s in each wind direction is having a negligible effect on the heat flux values measured.

## Conclusions

This study investigated numerically the influence of varying wind conditions on fire spread between two informal settlements dwellings (D1 and D2) separated by 1.0 m. The numerical model was initially evaluated against a still air benchmark experiment in the lab (no wind) for model benchmarking. The wind conditions changed in terms of speed (i.e., 6  m/s, 10  m/s, and 14 m/s) and direction (i.e., flowing to the North, South, East, and West) for the parametric study, while the two informal dwellings faced each other in such a way that an alley was established between them in the South-North direction. Although the East direction is in the same direction as the fire spread (i.e., flame projection from D1 to D2), the West direction cases had a somewhat faster ignition time. This is thought to be related to the observed back-step flow phenomenon in West direction cases, which created a low-pressure zone below the border of D2's ceiling, causing the external plume expelled from D1 openings to be 'drawn' towards D2 and impinge more significantly on it. Fire spread was highly delayed in situations when the wind stream travels through the alley between D1 and D2 in both directions (South and North), tilting the external plumes from D1 away from D2. Alley wind flow also had a significant impact on the time of flashover in D2, the time for fire to spread between the two dwellings, and the heat fluxes along the alley. For all directions, the overall heat transfer coefficient was found to be directly proportional to the wind speed and comparing different scenarios, the internal radiative heat transfer coefficient of one wall is sufficient to represent the overall heat transfer coefficient. Compared to all other scenarios, increasing the wind speed in circumstances where the wind reaches the window of both dwellings prior to the door, delays flashover occurrence (in both D1 and D2). Furthermore, it was found that even with initial fire spread to D2 with high wind speed flowing in the alley fire can be quenched in D2 before reaching flashover.
